# Hydrogen Sulfide Attenuates Atherosclerosis in a Partially Ligated Carotid Artery Mouse model via Regulating Angiotensin Converting Enzyme 2 Expression

**DOI:** 10.3389/fphys.2017.00782

**Published:** 2017-10-10

**Authors:** Yanjun Lin, Huasu Zeng, Lin Gao, Ting Gu, Changqian Wang, Huili Zhang

**Affiliations:** ^1^Department of Cardiology, Shanghai Ninth People's Hospital, Shanghai JiaoTong University School of Medicine, Shanghai, China; ^2^Department of Oral and Maxillofacial Pathology, Shanghai Ninth People's Hospital, Shanghai JiaoTong University School of Medicine, Shanghai, China

**Keywords:** hydrogen sulfide, atherosclerosis, ACE2, Ang-(1-7), Ang II

## Abstract

Hydrogen sulfide has been suggested to play an essential role in atherogenesis. There is a paucity of information about the association between H_2_S and angiotensin converting enzyme 2 (ACE2), a novel homolog of ACE. Therefore, the aim of the study was to explore the role of H_2_S in atherosclerosis with respect to ACE2 both *in vitro* and *in vivo*. Here, a murine model of acutely disturbed flow-induced atherosclerosis by left common carotid artery (LCA) partial ligation was utilized. We found that carotid partial ligation in high-fat fed apoE^−/−^ mice significantly inhibited endogenous H_2_S synthesis in LCA. Application of NaHS, an H_2_S donor considerably attenuated the severity of atherosclerosis with upregulating carotid expression of ACE2, thus converting pro-atherosclerotic angiotensin II (Ang II) to anti-atherosclerotic angiotensin 1-7 (Ang-(1-7)). The anti-atherosclerotic effect of NaHS was dramatically abolished by treatment with MLN-4760, an ACE2 inhibitor. In contrast, blockage of H_2_S formation by DL-propargylglycine exacerbated the burden of atherosclerotic plaques accompanied by inhibiting carotid expression of ACE2. At the cellular level, NaHS dose-dependently promoted the expression of ACE2 and conversion from Ang II to Ang-(1-7) in unstimulated or LPS-stimulated endothelial cells, thus exerting anti-inflammatory properties. The anti-inflammatory effect of NaHS was abrogated by pretreatment with DX600, a selective ACE2 inhibitor. In conclusion, these data provide direct evidences that endogenous H_2_S insufficiency exists in acute flow disturbance-induced atherosclerosis and that application of H_2_S may protect against atherosclerosis via upregulating ACE2 expression in endothelial cells.

## Introduction

Hydrogen sulfide (H_2_S) is recently considered to be a novel gaseous mediator, which is endogenously produced during cysteine metabolism mainly by two pyridoxal phosphate-dependent enzymes, cystathionine β-synthase (CBS) and cystathionine γ-lyase (CSE) (Renga, 2011). It has become clear that H_2_S exerts various effects in mammalian cardiovascular tissues. Endogenous H_2_S has been suggested to be involved in the pathogenesis of atherosclerosis (Lefer, 2007; Elsey et al., 2010). Wang et al. found for the first time the association between H_2_S and atherosclerosis in apoE^−/−^mice (Wang et al., 2009). The causative effect of H_2_S in atherosclerosis was further confirmed in CSE gene-deficient mice (Mani et al., 2013). Deletion of CSE gene in mice led to decreased H_2_S formation, elevated blood pressure and impaired endothelium-dependent vasorelaxation (Yang et al., 2008). As a result, CSE knockout mice fed a high cholesterol diet predisposed to develop early atherosclerotic lesions (Mani et al., 2013). Mice with both CSE and apoE gene knockout had more extensive atherosclerosis burden than those with either apoE or CSE knockout (Mani et al., 2013). Furthermore, various studies have investigated the precise mechanisms by which H_2_S hinders the development of atherosclerosis. H_2_S might protect vascular tissue from atherogenic damage by inhibiting vascular intimal proliferation, reducing adhesion molecules expression, suppressing oxidative stress and limiting foam cell formation (Laggner et al., 2007; Meng et al., 2007; Zhao et al., 2011). However, due to the complexity of the atherogenic process, the anti-atherogenic mechanism of H_2_S is still far from clear.

It is well-known that renin-angiotensin system (RAS) is a key modulator of cardiovascular function and plays essential roles in endothelial dysfunction and atherosclerosis (Dzau, 2005; Savoia and Schiffrin, 2006). Angiotensin-converting enzyme (ACE)/angiotensin II (Ang II)/angiotensin II type 1 receptor (AT1R) axis is the main pathway of RAS and contributes to the pathogenesis of atherosclerosis (Dzau, 2005; Savoia and Schiffrin, 2006). Recently, the understanding of RAS has been greatly expanded after a homolog of ACE, namely ACE2 was identified. Unlike ACE, ACE2 stimulates the degradation of Ang II into Ang-(1-7), an anti-inflammatory vasodilator and anti-trophic heptapeptide (Donoghue et al., 2000; Oudit et al., 2003; Danilczyk and Penninger, 2006), therefore exerting protective effect in atherosclerosis. Moreover, the anti-atherosclerotic effect of ACE2 has been proposed by overexpression of ACE2 in mice or rabbits, as characterized by an increase in tissue Ang-(1-7) and a decrease in tissue Ang II (Dong et al., 2008; Lovren et al., 2008). Overexpression of ACE2 promoted the stability of atherosclerotic lesions by suppressing macrophage infiltration, decreasing lipid deposition, increasing collagen content and lowering matrix metalloproteinase activity in plaques (Dong et al., 2008). However, ACE2-deficicency in LDLR^−/−^ or apoE^−/−^ mice exacerbated the development of atherosclerosis (Thomas et al., 2010; Thatcher et al., 2011). Thus, ACE2 may provide a therapeutic target in the treatment of atherosclerotic cardiovascular diseases.

In light of the importance of RAS in atherosclerosis, some studies explored the relationship between H_2_S metabolism and RAS. It was found that H_2_S improved endothelial function and myocardial remodeling via downregulating Ang II/AT1R pathway in renovascular hypertensive rats (Xue et al., 2015; Liu et al., 2017). Application of NaHS, an H_2_S donor, inhibited hyperglycemia-induced ACE-Ang II-AT1R activation in cultured renal mesangial cells and kidneys from diabetic rats (Xue et al., 2013). Previous studies have investigated the role of H_2_S in ACE-Ang II- AT1R axis, the classical pathway of RAS. However, there is little information about the link between H_2_S and ACE2 in cardiovascular system. Given this background, this study was designed to probe the possible effect of H_2_S on ACE2-Ang-(1-7) *in vitro* and *in vivo*. Our data indicate that deficiency of endogenous H_2_S formation accompanies the development and progression of disturbed flow-induced atherosclerosis. Supplement of H_2_S upregulated ACE2 expression and production of Ang-(1-7) in endothelial cells, resulting in attenuation of atherosclerosis.

## Materials and methods

### Cell culture

Human umbilical vein endothelial cells (HUVECs) were obtained from American Type Culture Collection (ATCC). Cells were cultured in Dulbecco's modified Eagle's medium (DMEM) supplemented with 10% fetal bovine serum (FBS) (Invitrogen, USA), 100 units/ml penicillin and 100 mg/ml streptomycin. Cells were plated in 6-well plates or 100-mm tissue culture dishes 1 day before experiments. Near-confluent cultures were starved overnight in medium containing 0.5% FBS before NaHS treatment or stimulation with LPS.

### Cell treatment

All treatments were performed in serum-free culture medium with penicillin and streptomycin. Cells were washed twice with serum-free culture medium and pre-incubated with saline or NaHS (50, 100, and 200 μM) for 24 h. Some cells were then stimulated with LPS (100 ng/ml) for 24 h in the continuous presence of NaHS. In the time course experiment, cells were pre-incubated with saline or NaHS (100 μM) for 0, 6, 24, or 48 h. For experiments using inhibitors, cells were pre-treated with DX600 (1 μM, an ACE2 inhibitor, Phoenix Pharmaceuticals; Pedersen et al., 2011) for 2 h before pre-incubation with NaHS (100 μM) for 24 h and subsequent stimulation with LPS (100 ng/ml) for 24 h.

### Animal model

**Figure 1:**
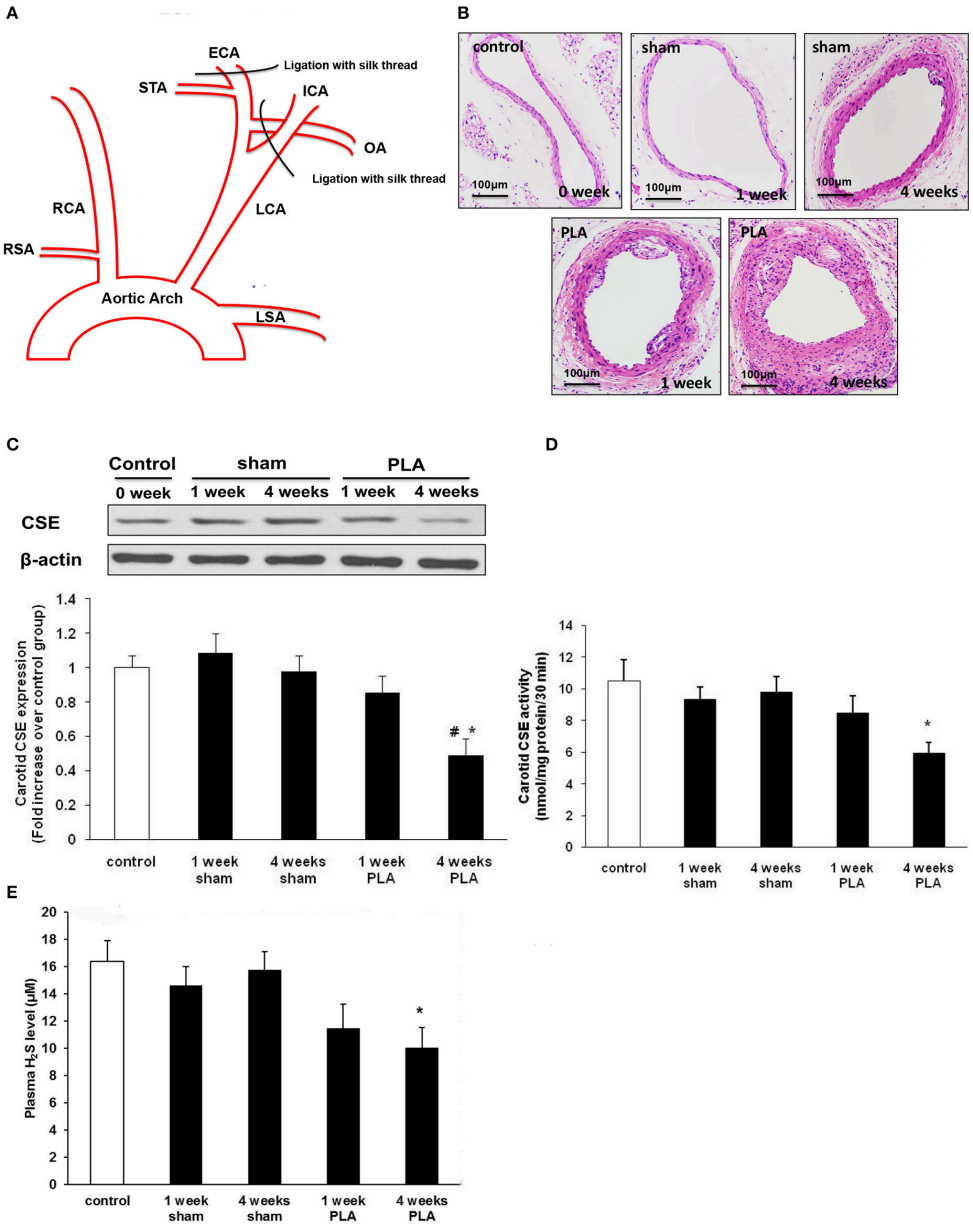
**(A)** Schematic diagram of low and oscillatory flow-induced atherosclerosis by partial ligation of LCA (PLA). Three branches of LCA, external carotid artery (ECA), internal carotid artery (ICA), and occipital artery (OA) were ligated with 6-0 silk suture, while leaving the superior thyroid artery (STA) intact. **(B)** Representative light microscopy images of hematoxylin and eosin-stained LCA cross sections were taken from high-fat fed apoE^−/−^ mice 1 or 4 weeks after PLA or sham operation. Scale bar for histological images = 100 μm. **(C–E)** Alterations in H_2_S biosynthesis during PLA induced atherosclerosis in high-fat fed apoE^−/−^ mice. CSE expression **(C)** and activity **(D)** in LCA, and plasma H_2_S level **(E)** were assayed at indicated time points (1 or 4 weeks after PLA or sham operation). Results shown are the mean ± SEM (*n* = 6 animals in each group). ^*^P < 0.05 for the comparison between mice 4 weeks after sham operation and mice 4 weeks after PLA. ^#^*P* < 0.05 for the comparison between mice 1 week after PLA and mice 4 weeks after PLA.

C57BL/6J male apoE^−/−^ mice were purchased from the Animal Center of the Beijing University, Beijing, China. The study was carried out in accordance with the Guidelines for the Care and Use of Laboratory Animals of the Shanghai JiaoTong University School of Medicine. The protocol was approved by the Committee on the Ethics of Animal Experiments of the Shanghai JiaoTong University School of Medicine (Permit Number: [2015]-117). At age of 8 weeks, mice were randomly assigned to partial ligation of left carotid artery (LCA) or sham operation, followed by being fed a high-fat diet for 4 weeks that contained 10% fat from lard and was supplemented with 2% (w/w) cholesterol. LCA was partially ligated as previously described (Sullivan, 2002; Nam et al., 2009) with slight modifications. In brief, mice were intrapeitoneally anesthetized by the mixture of xylazine (10 mg/kg) and ketamine (80 mg/kg). The neck was epilated and then disinfected with iodophor. LCA was exposed by a ventral midline incision (4–5 mm) in the neck. Except that the superior thyroid artery remained intact, left external carotid, internal carotid and occipital artery were ligated with 6–0 silk (Figure 1A). The skin was sutured and mice were then kept in a warm chamber until recovery. After LCA partial ligation or sham operation, mice were randomly given saline, NaHS (1 mg/kg/day, i.p.) or DL-propargylglycine (PAG, 10 mg/kg/day, i.p.). Some NaHS-treated mice were intraperitoneally given MLN-4760, a selective ACE2 inhibitor (0.5 mg/kg, daily, Millennium Pharmaceuticals) 2 weeks after initiation of NaHS treatment (Ye et al., 2012). Four weeks postligation, all the mice were sacrificed. Samples of carotid arteries and blood were collected and stored at −80°C. At the beginning and the end of the study, systolic blood pressure (SBP) was monitored by a tail cuff system (Blood Pressure Analysis System BP- 98AW monitor, Japan).

### Histological examination and masson staining

Anesthetized mice underwent left ventricle perfusion with buffered saline and 10% neutralized formalin at 100 mmHg. LCA was collected en bloc with the trachea and esophagus. LCA were embedded in paraffin. Fifteen serial sections (5 μm) were taken 500–1,000 μm proximal to the location of ligation. Five sections from each mouse were stained with hematoxylin and eosin. The staining were then examined by light microscopy (*n* = 6 mice in each group, objective lens magnification of × 20; eyepiece magnification of × 10). The lesions were quantified by Image J software (National Institutes of Health, USA) as described in the literatures (Lessner et al., 2002; Sullivan, 2002; Nam et al., 2009; Zhang et al., 2012, 2015). Measurement of intima and media of carotid arteries was acquired by tracing the border of the lumen and the internal and external elastic laminae (Figure S1). The mean area of the intima and media was measured by tracing multiple sections, and the ratio of the area of intima to media was calculated. Sections were also stained with Masson's trichrome to display collagen components in a given plaque. To calculate proportions of collagen content to neointima, we measured intimal lesion areas and Masson's trichrome positive blue areas in at least three sequential sections (Tasaki et al., 2013). Every morphological parameter was quantified by one investigator blinded for the treatment.

### Immunohistochemistry of ACE2

LCA were deparaffinized in xylene and rehydrated in aqueous solutions with decreasing alcohol content, followed by a wash in PBST (1 × PBS with 0.5% Tween 20, pH 7.4). Antigen retrieval was achieved by heating the slides in 10 mM sodium citrate (PH 6.0) at 95°C for 20 min. Slides were gradually cooled down at room temperature and washed in H_2_O and PBST. To inactivate endogenous peroxidase, sections were treated with 3% H_2_O_2_ for 15 min, followed by incubation with 10% normal goat serum for 20 min to block nonspecific staining. After incubation overnight at 4°C with primary antibody (rabbit anti-mouse ACE2 in a dilution of 1:75, Abcam, USA), slides were incubated with secondary antibody (HRP-conjugated, goat anti-rabbit IgG in a dilution of 1:200, Santa Cruz Biotechnology, USA) for 2 h at room temperature. Specific staining for ACE2 was developed by the reaction with 3, 3′-diaminobenzidine and counterstaining was applied with hematoxylin. The immunohistochemical staining was viewed by light microscopy (objective lens magnification of × 40; eyepiece magnification of × 10).

### Real-time RT-PCR

Total RNA from cells was extracted using Trizol ® reagent (Invitrogen, Carlsbad, CA, USA) according to the manufacturer's protocol. The concentration of isolated RNA was assayed by measuring the absorbance at 260 nm and the integrity of RNA was visualized by ethidium bromide staining of 18S and 28S on a denaturing agarose gel. One microgram RNA was reversely transcribed using iScript™ cDNA Synthesis Kit (Biorad, USA) at 25°C for 5 min, 42°C for 30 min, followed by 85°C for 5 min. The resulting cDNA was then used as a template for real time PCR amplification. The forward and reverse primers of ACE2, ACE, Mas, and β-actin gene were shown in Table 1. Real-time PCR was performed using Lightcycler 2.0 system (Roche Applied Science, USA). Relative expression of gene mRNA was analyzed using a comparative method described in the user bulletin. The data were calculated with 2^−ΔΔCT^ method and normalized to β-actin expression.

**Table 1:** Real time PCR primers.

**Genes**	**Primers**
ACE2	F: 5′-ACCCTTCTTACATCAGCCCTACTG-3′
	R: 5′-TGTCCAAAACCTACCCCACATAT-3′
ACE	F: 5′-CAGCTTCATCAT-CCAGTTCC-3′
	R: 5'-CCAGGAAGAG-CAGCAGCCAC-3′
Mas	F: 5′-ACAACACGGGCCTCTATCTG-3′
	R: 5′-CTCATGGGCATAGCGAAGAT-3′
β-actin	F: 5′-GGATGCAGAAGG AGATCACTG-3′
	R: 5′-CGATCCACACGGA GTACTTG-3′

### Western immunoblot

Cells (3 × 10^6^) were collected and lysated at 4°C using radioimmunoprecipitation assay lysis buffer. Tissues of LCA were homogenized on ice in radioimmunoprecipitation assay lysis buffer. Cell lysates or tissue homogenates were centrifuged at 14,000 g for 10 min at 4°C. Protein concentration in the soluble fraction was determined by Bradford method. Proteins (20 μg) were size-fractionated by 8% SDS-PAGE and transferred onto nitrocellulose membranes. The membranes were blocked for 2–3 h with 5% nonfat milk and then probed overnight at 4°C with rabbit polyclonal anti-ACE2 (1:500, molecular weight 97 kDa, Abcam, USA), β-actin antibodies (1:1,000, Santa Cruz Biotechnology, USA) and mouse monoclonal anti-CSE antibody (1:1,000, molecular weight 45 kDa, Abnova, Taiwan) respectively, followed by secondary antibody for 2 hs with a 1:2,000 dilution of HRP-conjugated, goat anti-rabbit IgG or goat anti-mouse IgG (Santa Cruz Biotechnology, USA). The blots were visualized using a standard enhanced chemiluminescence system.

### ELISA

MCP-1, TNF- α and IL-6 (Quantikine, R&D systems), as well as Ang-(1-7) and Ang II (Cloud-Clone Corp., USA) were assayed using ELISA kits according to the manufacturers' instructions. Results for the levels of Ang-(1-7) and Ang II in carotid arteries were expressed as pg/mg protein after correction for the protein concentration in tissue homogenates (determined using the Bradford assay).

### Measurement of plasma H_2_S

Ten percent Trichloroacetic acid (120 μl), 1% zinc acetate (60 μl), 20 μM N, N-dimethyl-p-phenylenediamine sulfate (40 μl) in 7.2 M hydrochloride acid and 30 μM FeCl_3_ (40 μl) in 1.2 M hydrochloride acid were mixed with plasma (120 μl) and distilled water (100 μl). After 10 min, the absorbance of the mixture was assayed by spectrophotometry at 670 nm (Tecan Systems Inc.). Plasma level of H_2_S was calculated using a standard curve of NaHS with a concentration from 3.125 to 100 μM.

### H_2_S synthesizing activity assay

H_2_S synthesizing activity in LCA was measured as described previously (Zhang et al., 2006). In brief, 4.5% w/v tissue homogenate (430 μl) in 100 mM potassium phosphate buffer (pH 7.4) was mixed with 20 mM L-cysteine (20 μl), 2 mM pyridoxyal 5′-phosphate (20 μl) and saline (30 μl). The reaction started in tightly sealed tubes after tubes were transferred from ice to water bath at 37°C. After incubation for half an hour, 1% zinc acetate (250 μl) was added and trapped the evolved H_2_S, followed by 10% trichloroacetic acid (250 μl) to cease the reaction. Afterward, 20 μM N, N-dimethyl-p-phenylenediamine sulfate (133 μl) in 7.2 M hydrochloride acid was added, immediately followed by 30 μM FeCl3 (133 μl) in 1.2 M hydrochloride acid. The absorbance of the resulting mixture was assayed by spectrophotometry at 670 nm (Tecan Systems Inc). H_2_S concentration was calculated using a standard curve of NaHS with a range from 3.125 to 100 μM. Results were expressed as nmoles H_2_S produced per mg protein in tissue homogenates (determined using the Bradford assay).

### Statistics

The data were expressed as mean ± SEM. The significance of differences among groups was evaluated by analysis of variance (ANOVA) with post-hoc Tukey's test when comparing three or more groups. The significance of differences between two groups was evaluated by *T*-test. A *P* < 0.05 was regarded as statistically significant.

## Results

### Alterations of endogenous H_2_S synthesis during disturbed flow-induced atherosclerosis

Atherosclerosis is known to be closely associated with disturbed flow characterized by low and oscillatory shear stress (Sullivan, 2002; Pedersen et al., 2011). However, studies directly linking H_2_S to disturbed flow condition in atherogenesis are lacking. Here, we investigated the alterations of H_2_S metabolism in a murine model of acutely disturbed flow-induced atherosclerosis by partial carotid ligation (Figure 1A). ApoE^−/−^ mice underwent either LCA partial ligation or sham operation and then were fed a high-fat diet for 1 or 4 weeks. By the first week, LCA showed slight evidences of atherosclerotic lesions as determined by H&E staining (Figure 1B). By 4 weeks, LCA developed accelerated atherosclerosis (Figure 1B). However, only minor or no lesions were observed in carotid arteries isolated from mice fed a high-fat diet for 1 or 4 weeks after sham operation (Figure 1B).

Then, we examined the time-dependent alterations of carotid H_2_S bio-synthesis in disturbed flow-induced atherosclerosis. As shown in Figures 1C,D, H_2_S synthesizing activity and CSE expression in LCA decreased in a time-dependent fashion, with a significant reduction 4 weeks after ligation. Furthermore, plasma H_2_S concentration gradually declined in a time-dependent manner (Figure 1E). There was a statistically significant reduction in plasma H_2_S level by 4 weeks after ligation. These data suggest both local and systemic H_2_S insufficiency in LCA partial ligation induced atherosclerosis. In another word, acute flow disturbance induced by partial ligation inhibited carotid CSE expression and CSE activity, thus resulting in an evident decline in production of endogenous H_2_S.

### Alterations of ACE2 in carotid arteries during disturbed flow-induced atherosclerosis

**Figure 2:**
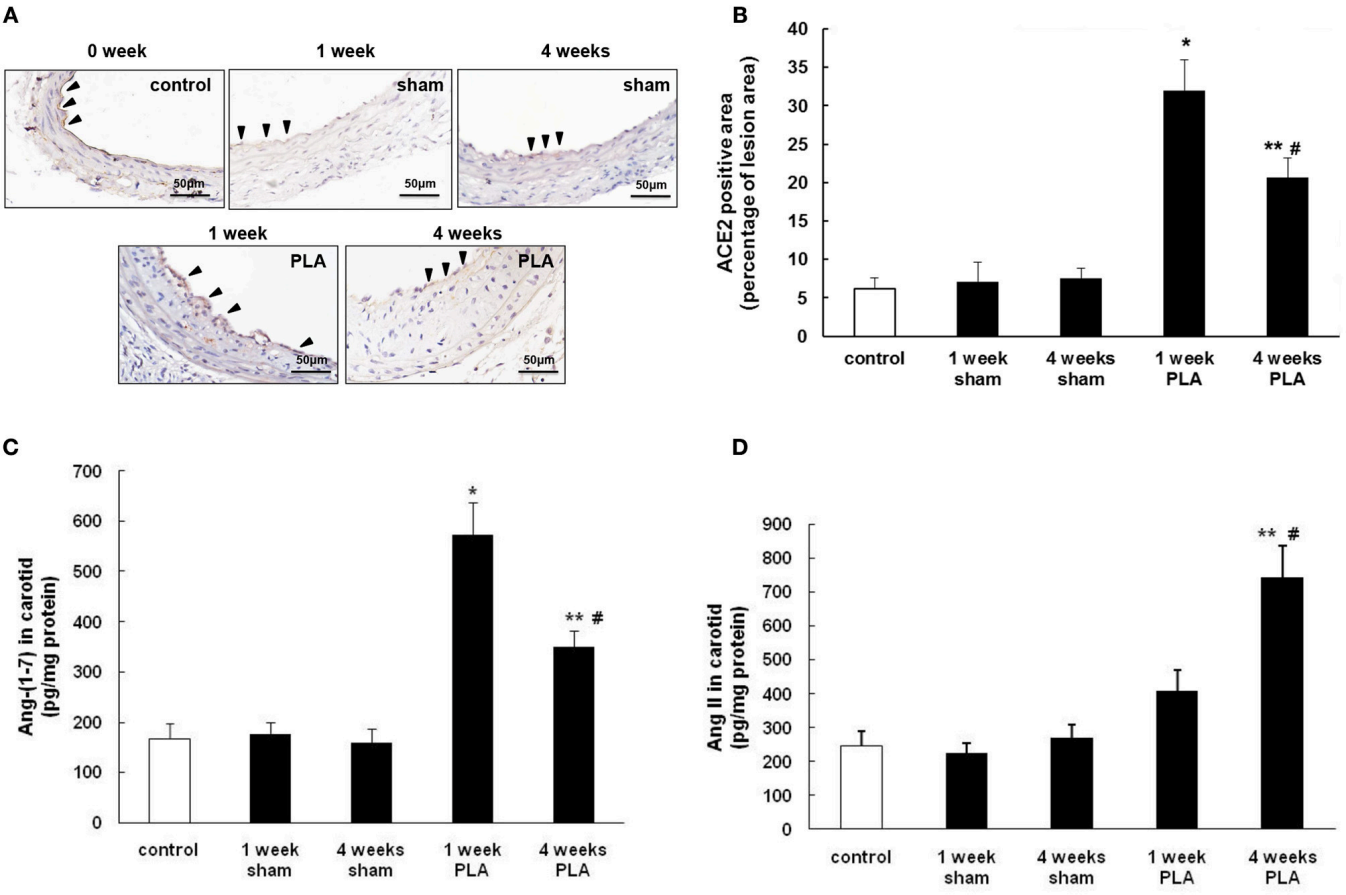
Alterations in ACE2 expression during PLA induced atherosclerosis in high-fat fed apoE^−/−^ mice. **(A)** Representative immunohistochemical images of LCA cross sections were taken from high-fat fed apoE^−/−^ mice 1 or 4 weeks after PLA or sham operation. Scale bar for histological images = 50 μm. **(B)** LCA sections from mice 1 or 4 weeks after PLA or sham operation were quantified immunohistochemically for ACE2 positive staining. Levels of Ang-(1-7) **(C)** and Ang II **(D)** in LCA were assayed by ELISA at indicated time points (1 or 4 weeks after PLA or sham operation). Results shown are the mean ± SEM (*n* = 6 animals in each group). Arrowheads represented positive staining for ACE2 in endothelial cells. ^*^P < 0.05 for the comparison between mice 1 week after sham operation and mice 1 week after PLA. ^**^P < 0.05 for the comparison between mice 4 weeks after sham operation and mice 4 weeks after PLA. ^#^*P* < 0.05 for the comparison between mice 1 week after PLA and mice 4 weeks after PLA.

Next, we explored the alterations of carotid ACE2 over time in disturbed flow-induced atherosclerosis (Figure 2A). Immunostaining showed that ACE2 was finely expressed in vascular endothelial cells from normal mice. By 1 week postligation, expression of ACE2 in LCA endothelim was significantly upregulated. However, by 4 weeks, the endothelial expression of ACE2 dramatically decreased with the progression of atherosclerosis (Figures 2A,B). Four weeks after ligation, LCA endothelium exhibited less intense staining with ACE2 than 1 week after ligation. As a result, the level of Ang-(1-7) in LCA was high at the beginning of ligation and then gradually reduced in a time dependent manner (Figure 2C). The level of Ang II in LCA changed oppositely (Figure 2D). These data indicate that disturbed flow in carotid arteries initially induced the endothelial expression of ACE2 in an attempt to inhibit the initiation of atherosclerosis. However, with the development and progression of atherosclerosis, endothelial ACE2 expression was inhibited, therefore shifting a balance from anti-atherosclerotic Ang-(1-7) to pro-atherosclerotic Ang II.

### Effect of H_2_S on disturbed flow-induced atherosclerosis

In the time course study, we found that the biosynthesis of H_2_S significantly decreased 4 weeks after PLA whereas the carotid ACE2 expression started rising 1 week after PLA (Figures 1, 2). This finding suggested that H_2_S seems to play a part in regulating ACE2 expression in the advanced stage of atherosclerosis (4 weeks after PLA) but not in the early phase of atherosclerosis (1 week after PLA). In addition to H_2_S, there are some other factors contributing to ACE2 expression in different stages of atherosclerosis. Here, we investigated the effect of H_2_S on carotid ACE2 expression 4 weeks after PLA.

**Figure 3:**
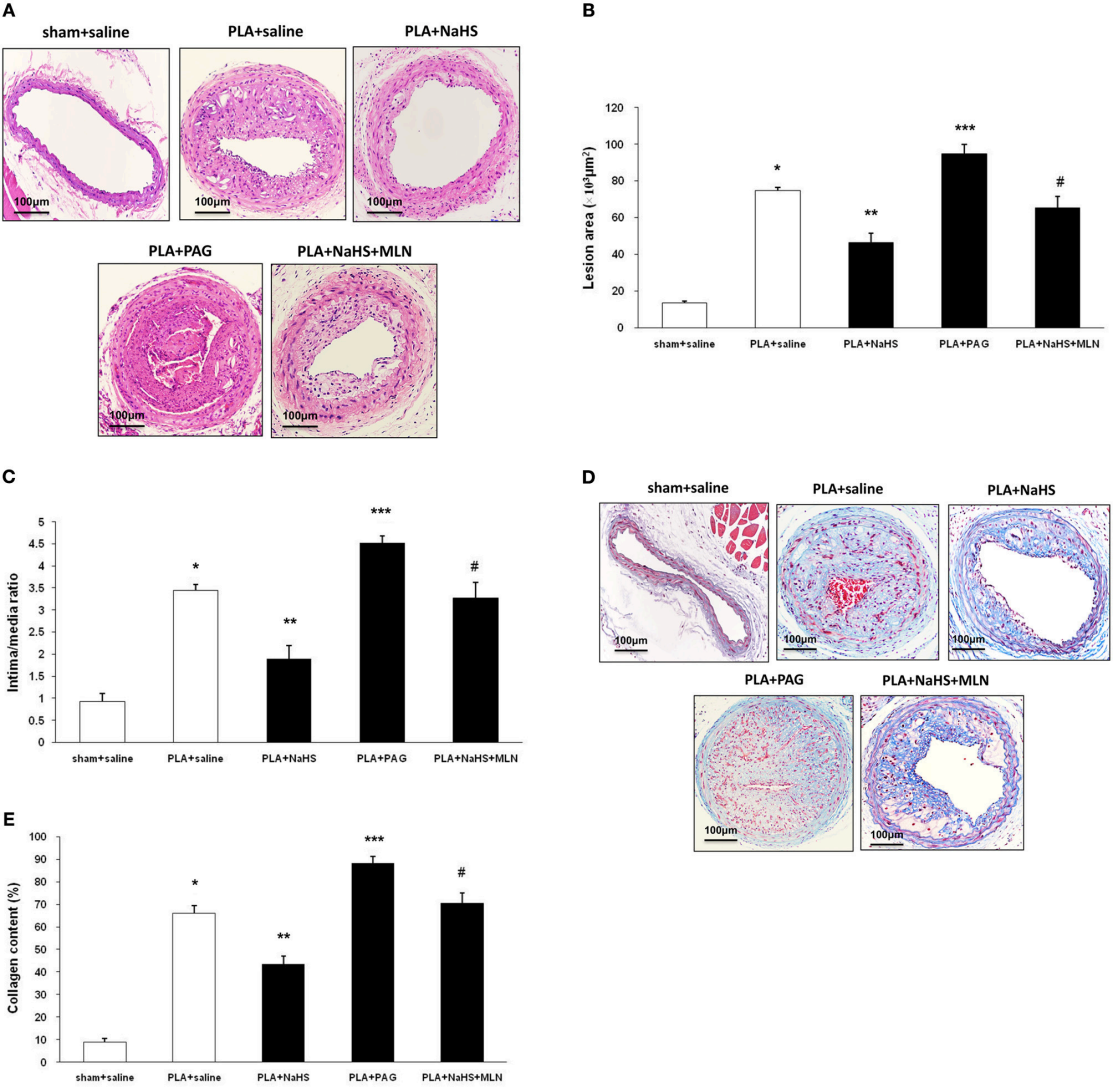
Effect of NaHS or PAG on PLA-induced atherosclerosis in LCA. Representative light microscopy images of hematoxylin and eosin-stained **(A)** or Masson trichrome-stained **(D)** LCA cross sections were taken from sham-operated mice with saline, PLA mice with NaHS, PLA mice with PAG, or PLA mice with NaHS and MLN (intervention with MLN-4760 for 14 days). Scale bar for histological images = 100 μm. Lesion area of intima **(B)**, intima/media ratio **(C)** and collagen deposits **(E)** in the neointimal hyperplasia in LCA were measured as described in Materials and Methods. Results shown are the mean ± SEM (*n* = 6 animals in each group). ^*^P < 0.05 for the comparison between sham+saline and PLA+saline. ^**^P < 0.05 for the comparison between PLA+saline and PLA+NaHS. ^***^P < 0.05 for the comparison between PLA+saline and PLA+PAG. ^#^*P* < 0.05 for the comparison between PLA+NaHS and PLA+NaHS+MLN.

NaHS (1 mg/kg/day, i.p.), DL-propargylglycine (PAG, 10 mg/kg/day, i.p.) or saline was randomly given to mice after partial ligation. After 4 weeks, the extent of atherosclerotic lesions was assessed by histological analysis (Figure 3A). Histological analysis showed that NaHS treatment significantly impeded the plaque development, as characterized by alleviated neointimal hyperplasia and less atherosclerotic lesions in LCA (Figures 3B,C). On the other hand, inhibition of H_2_S formation by PAG induced advanced atherosclerosis, as evidenced by more severe neointimal hyperplasia and more obvious narrowness in LCA (Figures 3B,C). Masson's staining revealed that NaHS reduced the amount of collagen deposits whereas PAG enhanced hyperplasia of collagen fibers in neointimal lesions (Figures 3D,E). In addition, NaHS or PAG had negligible effect on body weight, plasma lipid profiles, and blood pressure (Table 2).

**Table 2:** Effects of treatment with NaHS on blood pressure and plasma lipids.

	**Body Weight (g)**	**Systolic Blood Pressure (mmHg)**	**Total Cholesterol (mmol/L)**	**Triglycerides (mmol/L)**	**High-density Lipoprotein (mmol/L)**	**Low-density Lipoprotein (mmol/L)**
sham +saline	33.11 ± 5.61	128.74 ± 17.27	10.21 ± 2.51	1.87 ± 0.66	1.97 ± 0.71	7.21 ± 2.05
PLA+saline	31.65 ± 3.27	125.86 ± 15.26	11.43 ± 3.45	1.90 ± 0.67	2.02 ± 0.63	7.32 ± 3.68
PLA+NaHS	30.56 ± 3.36	109.23 ± 25.26	10.78 ± 2.96	2.01 ± 0.85	1.84 ± 0.77	8.10 ± 3.18
PLA+PAG	29.06 ± 4.85	134.45 ± 21.88	11.19 ± 3.09	2.14 ± 0.99	2.16 ± 0.63	7.65 ± 2.77
PLA+NaHS+MLN	28.75 ± 5.86	112.06 ± 23.32	11.52 ± 3.18	2.34 ± 1.04	2.08 ± 0.69	8.19 ± 2.74

### Effect of H_2_S on ACE2-Ang-(1-7) expression in atherosclerosis

**Figure 4:**
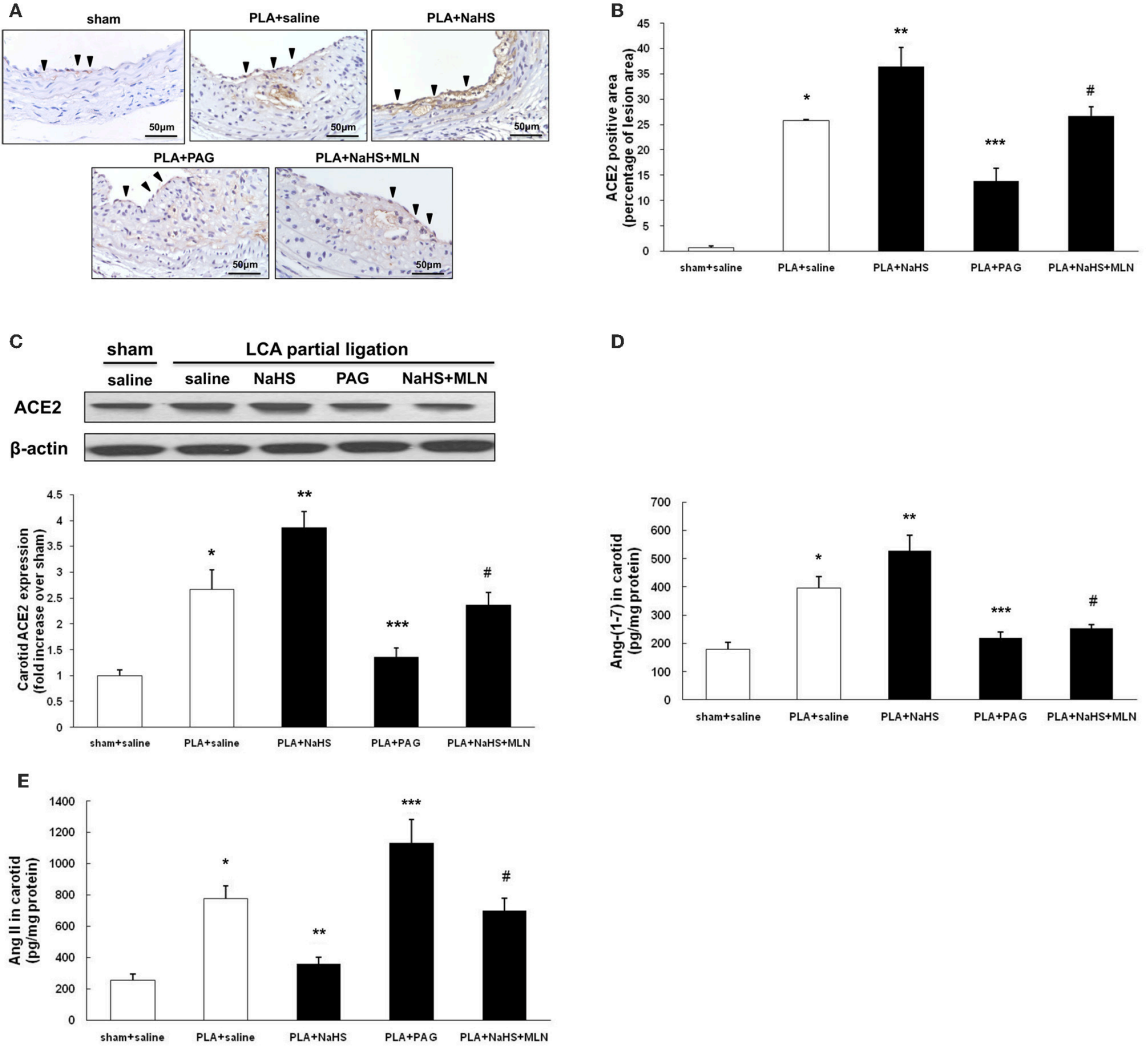
Effect of NaHS or PAG on ACE2 expression in LCA from mice with PLA-induced atherosclerosis. **(A)** Representative immunohistochemical images of LCA cross sections were taken from sham-operated mice with saline, PLA mice with NaHS, PLA mice with PAG, or PLA mice with NaHS and MLN (intervention with MLN-4760 for 14 days). Scale bar for histological images = 50 μm. **(B)** LCA sections were quantified immunohistochemically for ACE2 positive staining. **(C)** ACE2 expression in LCA was measured by Western blot as described in Materials and Methods. Levels of Ang (1-7) **(D)** and Ang II **(E)** in LCA were assayed by ELISA. Results shown are the mean ± SEM (*n* = 6 animals in each group). Arrowheads represented positive staining for ACE2 in endothelial cells. ^*^P < 0.05 for the comparison between sham+saline and PLA+saline. ^**^P < 0.05 for the comparison between PLA+saline and PLA+NaHS. ^***^P < 0.05 for the comparison between PLA+saline and PLA+PAG. ^#^*P* < 0.05 for the comparison between PLA+NaHS and PLA+NaHS+MLN.

Immunohistochemistry revealed that supplement with NaHS enhanced the expression of ACE2 in endothelial cells whereas PAG inhibited it in atherosclerotic lesions (Figures 4A,B). Then, we used Western blot to quantify the ACE2 expression level in LCA. Consistent with the findings obtained by immunostaining, NaHS significantly upregulated carotid ACE2 expression while PAG downregulated it (Figure 4C). As a result, NaHS notably promoted the local production of Ang (1-7) in LCA but PAG inhibited it (Figure 4D). The carotid level of Ang II was blunted by NaHS while it was raised by PAG (Figure 4E).

In order to reinforce the influence of H_2_S on ACE2-Ang-(1-7), MLN-4760, a selective and potent inhibitor against mouse ACE2 was applied (Ye et al., 2012). As shown in Figures 4D,E, blockage of ACE2 activity by MLN-4760 significantly reversed the elevation of carotid Ang-(1-7) level and the decline of carotid Ang II level induced by NaHS. The anti-atherosclerotic property of NaHS was also significantly abolished by treatment with MLN-4760 (Figures 3A,D).

### Effect of H_2_S on ACE2 in endothelial cells

**Figure 5:**
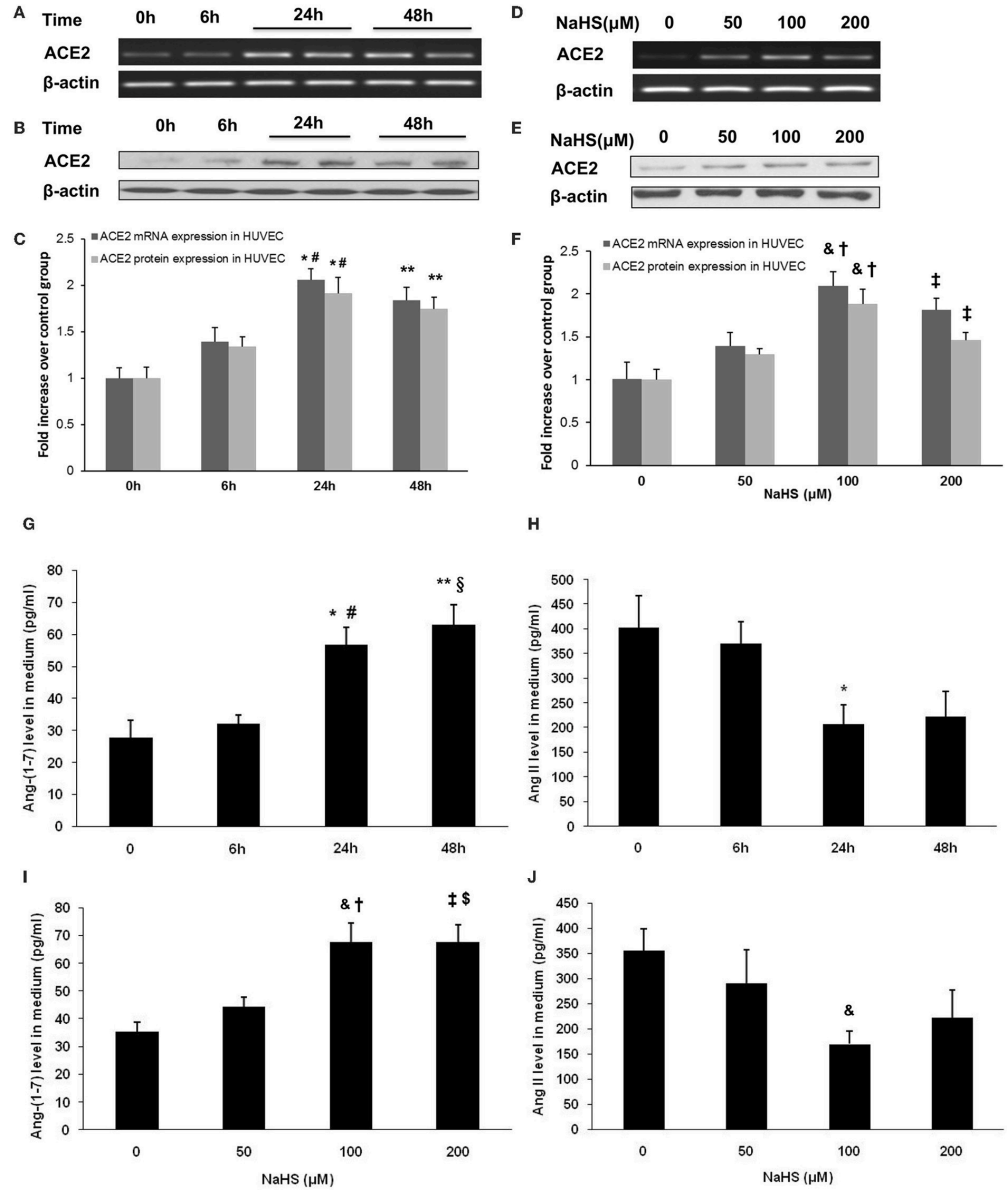
Effect of NaHS on ACE2 and Ang-(1-7) expression in HUVECs. In the time course study, cells were pre-incubated with saline or NaHS (100 μM) for 0, 6, 24 or 48 h **(A–C,G,H)**. In the dose ranging study, cells were pre-incubated with saline or NaHS (50, 100, and 200 μM) for 24 h **(D–F,I,J)**. ACE2 mRNA **(A,C,D,F)** and protein expression **(B,C,E,F)** were analyzed by real time PCR and Western blot respectively. Levels of Ang (1-7) **(G,I)** and Ang II **(H,J)** in culture medium were assayed by ELISA. The data are means ± SEM of at least three independent experiments. ^*^P < 0.05 for the comparison between control at baseline and HUVECs treated with NaHS for 24 h. ^**^P < 0.05 for the comparison between control at baseline and HUVECs treated with NaHS for 48 h. ^#^*P* < 0.05 for the comparison between HUVECs treated with NaHS for 6 h and HUVECs treated with NaHS for 24 h. ^§^*P* < 0.05 for the comparison between HUVECs treated with NaHS for 6 h and HUVECs treated with NaHS for 48 h. ^&^*P* < 0.05 for the comparison between control at baseline and HUVECs treated with NaHS at a concentration of 100 μM. ^†^*P* < 0.05 for the comparison between HUVECs treated with NaHS at a concentration of 50 μM and HUVECs treated with NaHS at a concentration of 100 μM. ^‡^*P* < 0.05 for the comparison between control at baseline and HUVECs treated with NaHS at a concentration of 200 μM. ^$^*P* < 0.05 for the comparison between HUVECs treated with NaHS at a concentration of 50 μM and HUVECs treated with NaHS at a concentration of 200 μM.

**Figure 6:**
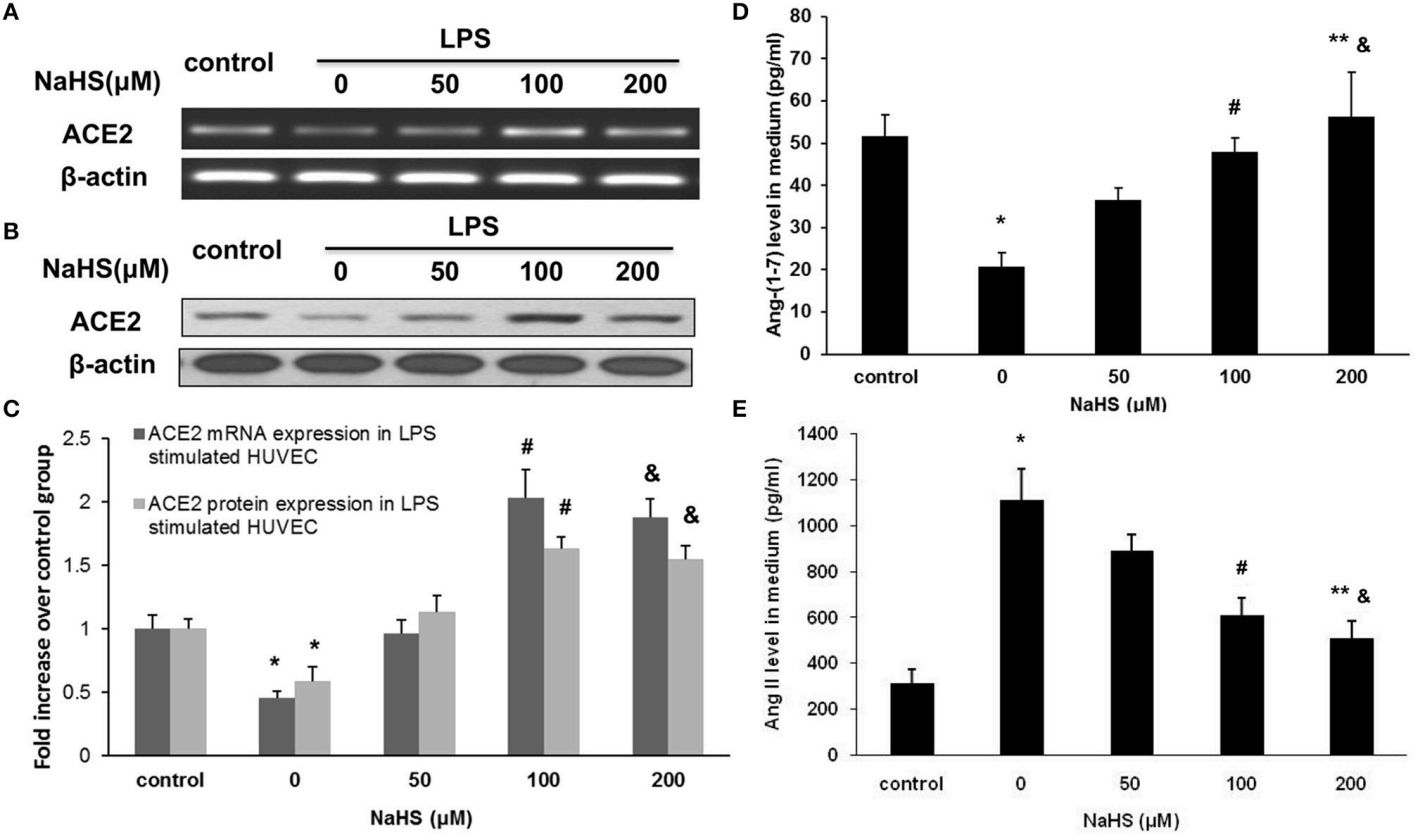
Effect of NaHS on ACE2 and Ang-(1-7) expression in HUVECs stimulated with LPS (100 ng/ml). HUVECs were pre-incubated with saline or NaHS (50, 100, and 200 μM) for 24 h and subsequently stimulated with saline or LPS (100 ng/ml) for 24 h. ACE2 mRNA **(A,C)** and protein **(B,C)** expression were analyzed by real time PCR and Western blot respectively. Levels of Ang (1-7) **(D)** and Ang II **(E)** in culture medium were assayed by ELISA. The data are means ± SEM of at least three independent experiments. ^*^P < 0.05 for the comparison between control at baseline and LPS-stimulated HUVECs treated with saline. ^#^*P* < 0.05 for the comparison between LPS-stimulated HUVECs treated with saline and LPS-stimulated HUVECs treated with NaHS at a concentration of 100 μM. ^&^*P* < 0.05 for the comparison between LPS-stimulated HUVECs treated with saline and LPS-stimulated HUVECs treated with NaHS at a concentration of 200 μM. ^**^P < 0.05 for the comparison between LPS-stimulated HUVECs treated with NaHS at a concentration of 50 μM and LPS-stimulated HUVECs treated with NaHS at a concentration of 200 μM.

Since ACE2 is highly expressed in carotid endothelium, we sought to examine the effect of H_2_S on ACE2-Ang-(1-7) in HUVECs. We found that NaHS time-dependently (Figures 5A–C) and dose-dependently (Figures 5D–F) increased the mRNA and protein expression of ACE2 in HUVECs. Subsequently, NaHS enhanced the production of Ang-(1-7) (Figures 5G,I) but decreased the level of Ang II (Figures 5H,J) in a time-dependent and dose-dependent manner. Moreover, similar effect of NaHS was observed in LPS-stimulated HUVECs (Figure 6). LPS at a concentration of 100 ng/ml significantly downregulated the expression of ACE2 and Ang-(1-7) but increased the level of Ang II in HUVECs. The inhibition of endothelial ACE2-Ang-(1-7) expression induced by LPS was reversed by NaHS in a dose dependent manner (Figure 6). NaHS also dose-dependently reduced the level of Ang II in LPS-stimulated HUVECs (Figure 6E). As Mas receptor is a functional receptor for Ang-(1-7) (Santos et al., 2003), we evaluated the effect of NaHS on the mRNA expression of Mas in HUVECs. NaHS did not alter the mRNA expression of Mas in unstimulated or LPS-stimulated HUVECs (Figure S2A). In addition, we examined the effect of H_2_S on ACE, the classical component of RAS in HUVECs. After treatment with NaHS, the mRNA expression level of ACE remained unchangeable in unstimulated or LPS-stimulated HUVECs (Figure S2B).

### H_2_S inhibits the production of cytokines and chemokine in endothelial cells by an ACE2 dependent mechanism

**Figure 7:**
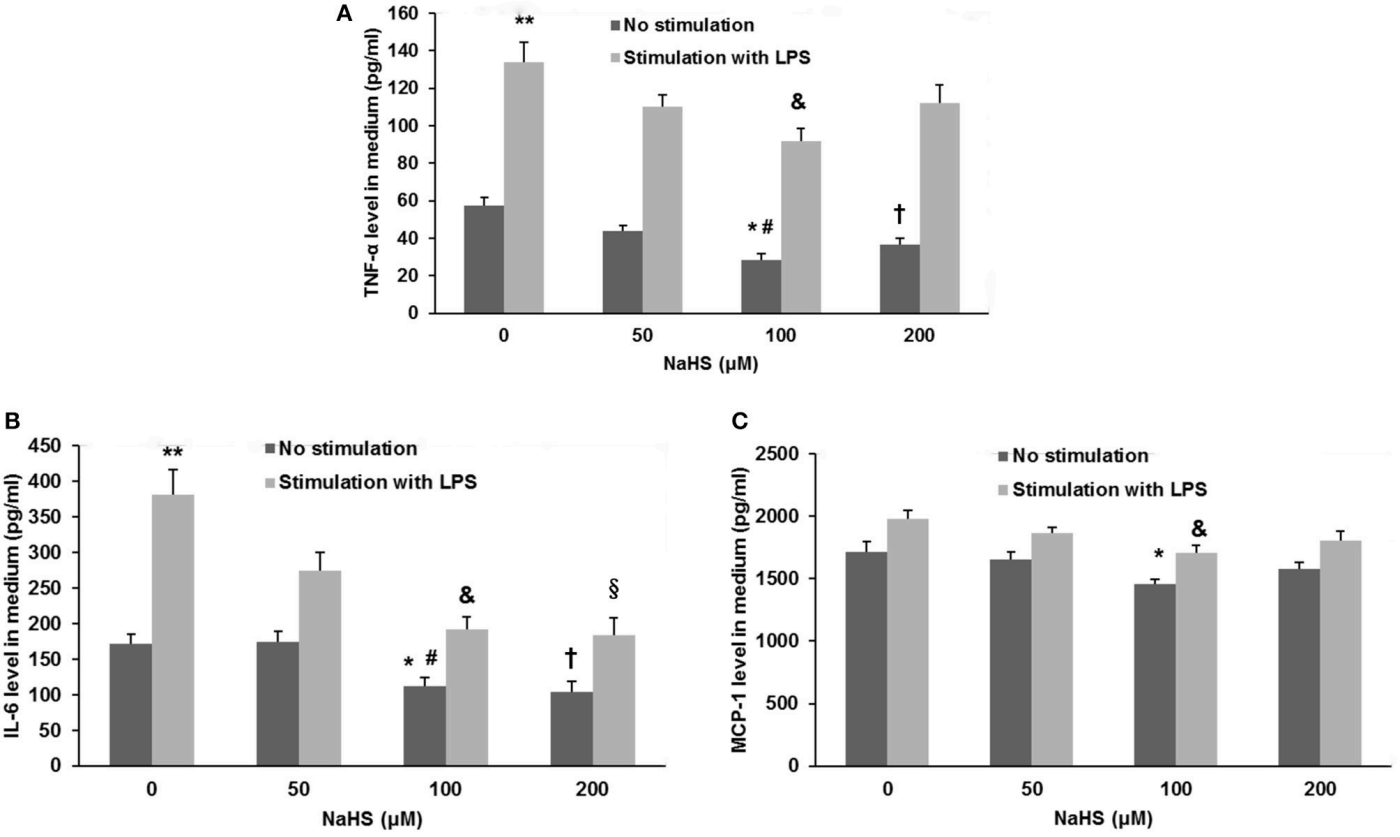
Effect of NaHS on cytokine and chemokine production in unstimulated or LPS-stimulated HUVECs. HUVECs were pre-incubated with saline or NaHS (50, 100, and 200 μM) for 24 h and subsequently stimulated with saline or LPS (100 ng/ml) for 24 h. Production of cytokines (TNF-α, IL-6) **(A,B)** and chemokine (MCP-1) **(C)** was assayed by ELISA. The data are means ± SEM of at least three independent experiments. ^*^P < 0.05 for the comparison between unstimulated HUVECs treated with saline and unstimulated HUVECs treated with NaHS at a concentration of 100 μM. ^#^*P* < 0.05 for the comparison between unstimulated HUVECs treated with NaHS at a concentration of 50 μM and unstimulated HUVECs treated with NaHS at a concentration of 100 μM. ^†^*P* < 0.05 for the comparison between unstimulated HUVECs treated with saline and unstimulated HUVECs treated with NaHS at a concentration of 200 μM. ^**^P < 0.05 for the comparison between unstimulated HUVECs treated with saline and LPS-stimulated HUVECs treated with saline. ^&^*P* < 0.05 for the comparison between LPS-stimulated HUVECs treated with saline and LPS-stimulated HUVECs treated with NaHS at a concentration of 100 μM. ^§^*P* < 0.05 for the comparison between LPS-stimulated HUVECs treated with saline and LPS-stimulated HUVECs treated with NaHS at a concentration of 200 μM.

**Figure 8:**
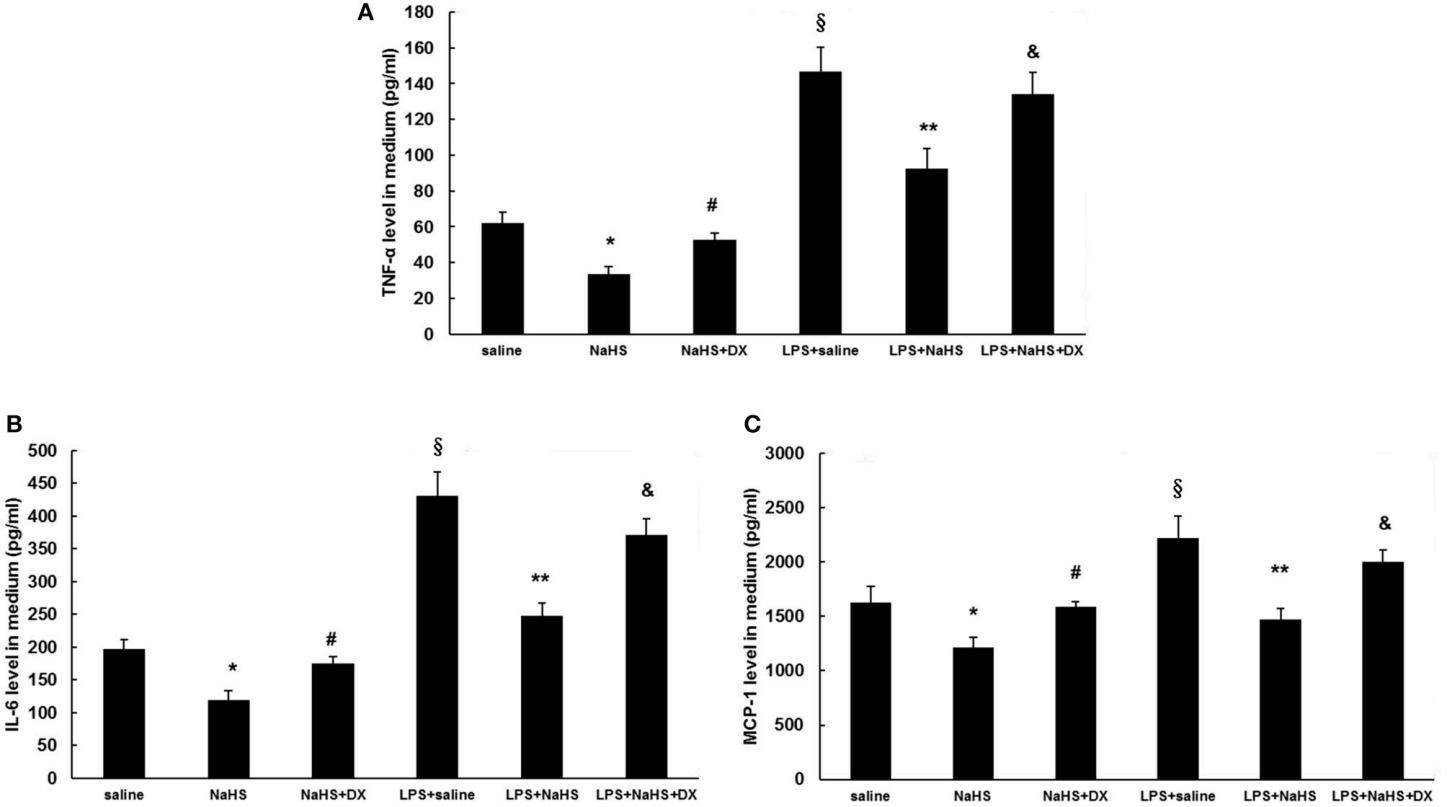
Effect of pretreatment with DX600, a selective ACE2 antagonist on suppressing the production of cytokines and chemokine induced by NaHS in unstimulated or LPS-stimulated HUVECs. Before pre-incubation with NaHS (100 μM) for 24 h, HUVECs were pre-treated with DX600 (1 μM) for 2 h. Then the cells were stimulated with saline or LPS (100 ng/ml) for 24 h. Production of cytokines (TNF-α, IL-6) **(A,B)** and chemokine (MCP-1) **(C)** was assayed by ELISA. The data are means ± SEM of at least three independent experiments. ^*^P < 0.05 for the comparison between control at baseline and unstimulated HUVECs treated with NaHS. ^#^*P* < 0.05 for the comparison between unstimulated HUVECs treated with NaHS and unstimulated HUVECs treated with NaHS and DX600. ^§^*P* < 0.05 for the comparison between control at baseline and LPS-stimulated HUVECs treated with saline. ^**^P < 0.05 for the comparison between LPS-stimulated HUVECs treated with saline and LPS-stimulated HUVECs treated with NaHS. ^&^*P* < 0.05 for the comparison between LPS-stimulated HUVECs treated with NaHS and LPS-stimulated HUVECs treated with NaHS and DX600.

As shown in Figure 7, the production of TNF-α, IL-6, and MCP-1 was dose-dependently repressed by NaHS in either unstimulated or LPS-stimulated HUVECs. The inhibitory effect of NaHS was significantly blunted by DX600, a selective ACE2 inhibitor (Nam et al., 2009), suggesting that H_2_S may inhibit endothelial activation through an ACE2-dependent mechanism (Figure 8).

In addition to endothelial cells, positive immunostaining for ACE2 in atherosclerotic plaques was also present in foam cells and macrophages (Figure 4A). H_2_S has been shown to be a potent regulator of monocyte/ macrophage activation (Zhi et al., 2007; Zhang et al., 2012), which is central to the pathogenesis of atherosclerosis. Therefore, we explored whether H_2_S would affect the activation of macrophages via ACE2-Ang-(1-7). As shown in Figure S3, NaHS significantly inhibited the production of TNF-α and MCP-1 in unstimulated or LPS-stimulated RAW264.7 cells. The anti-inflammatory property of NaHS was not reversed by pretreatment with DX 600, suggesting that H_2_S likely modulates macrophage functions through other pathways but not ACE2.

## Discussion

It is well established that atherosclerosis develops preferentially at particular sites in branched or curved arteries, which expose endothelial cells to disturbed flow characterized by low and oscillatory wall shear stress, even if there are various risk factors such as smoking, hyperlipidemia, diabetes and hypertension (Ku et al., 1985; VanderLaan et al., 2004). The present study utilized partial carotid ligation in high-fat fed apoE^−/−^ mice as an animal model of acutely disturbed flow-induced atherosclerosis and then investigated the role of H_2_S in it. To the best of our knowledge, this is the first study to explore the alterations of H_2_S biosynthesis in disturbed flow-induced atherosclerosis. We found that partial carotid ligation inhibited local CSE expression and CSE activity, thus resulting in an evident decline in endogenous production of H_2_S. Consistent with our findings, deficit of H_2_S synthesis was also obtained in fat-fed apoE^−/−^ mice or balloon injury induced neointimal hyperplasis (Meng et al., 2007; Wang et al., 2009; Zhang et al., 2012; Mani et al., 2013).

The biological importance of H_2_S in disturbed flow-induced atherosclerosis is further underlined by application of NaHS to manipulate endogenous H_2_S deficiency. NaHS treatment significantly reduced LCA atherosclerotic burden and impeded the progression of atherosclerosis. Furthermore, abolishing systemic H_2_S by PAG aggravated the extent of atherosclerosis in carotid arteries. These interesting findings highlight the potential role of H_2_S in the pathogenesis of atherosclerosis induced by low and oscillatory shear stress.

It is well documented that ACE2 plays a permissive role in reducing atherosclerosis. ACE2 is thought to counterbalance ACE by degrading pro-atherosclerotic Ang II to the putative protective peptide, Ang-(1-7) (Oudit et al., 2003; Dong et al., 2008; Lovren et al., 2008; Thomas et al., 2010; Thatcher et al., 2011). The anti-atherosclerotic properties of ACE2 were also evaluated in disturbed flow induced-atherosclerosis. We found that ACE2 was predominantly expressed in carotid endothelial cells in normal mice and that the expression of ACE2 significantly increased 1 week after partial ligation. With the progression of atherosclerosis, the expression level of ACE2 dramatically decreased. As a result, the level of Ang-(1-7) in LCA gradually reduced in a time dependent manner although it was high at the beginning of ligation. The level of Ang II in LCA changed oppositely. These data suggest that disturbed flow in carotid arteries initially upregulated the expression of ACE2 in an attempt to inhibit the initiation of atherosclerosis. However, with the development and progression of atherosclerosis, ACE2 expression decreased, thus shifting the balance from anti-atherosclerotic Ang-(1-7) to pro-atherosclerotic Ang II microenvironment. Consistent with our findings, expression of ACE2 mRNA and protein was observed in early and advanced human carotid atherosclerotic lesions (Sluimer et al., 2008). Overexpression of ACE2 by gene transfer attenuated the progression of atherosclerotic lesions in a rabbit model of atherosclerosis or mouse studies (Dong et al., 2008; Lovren et al., 2008). Knockout ACE2 gene in LDLR^−/−^ or apoE^−/−^ mice increased the development of atherosclerosis in aortic arch and sinus (Thomas et al., 2010; Thatcher et al., 2011). A meta-analysis involving 11,051 subjects suggests that genetic variants in ACE2 gene might have a potential effect on ACE2 activity, ACE2 level or Ang-(1-7) production and that ACE2 gene polymorphism may be a genetic risk factor for essential hypertension (Lu et al., 2012).

Furthermore, the association between H_2_S and ACE2 in atherosclerosis was explored in the present study. We found that application of exogenous H_2_S reversed partial ligation induced downregulation of ACE2 and Ang-(1-7) in LCA while blockage of H_2_S synthesis by PAG significantly aggravated it. The local level of Ang II was reduced by NaHS, but raised by PAG. Similar observations were obtained in cellular experiments. NaHS dose-dependently and time-dependently increased the expression of ACE2 in unstimulated or LPS-stimulated HUVECs. As a result, NaHS enhanced the production of Ang-(1-7) but decreased the level of Ang II. On the other hand, NaHS significantly inhibited the production of TNF-α, IL-6 and MCP-1 in unstimulated or LPS-stimulated HUVECs. The anti-inflammatory effect of NaHS in HUVECs was blunted by DX600. The anti-atherosclerotic benefit of NaHS was also abrogated by treatment with MLN-4760. Taken together, our findings provide solid evidences proposing that H_2_S plays a critical role in modulating endothelial ACE2 expression and promoting the cleavage of pro-atherosclerotic Ang II to anti-atherosclerotic Ang-(1-7), thereby impeding the development and progression of atherosclerosis. However, the precise mechanism by which H_2_S regulates the expression of ACE2 in endothelia cells during the initiation and progression of atherosclerosis remains elusive.

Peroxisome proliferator-activated receptors (PPAR) are nuclear receptors and function as transcription factors. PPARα has been suggested to affect the signaling pathway of RAS (Banks and Oyekan, 2008; Ibarra-Lara et al., 2010). Stimulation of PPARα with clofibrate favored ACE2/Ang-(1–7)/ATR2 axis in aortic coarctation-induced hypertensive rats as evidenced by enhancing Ang-(1-7) and ACE2 expression in the heart (Ibarra-Lara et al., 2016). Recent studies found that H_2_S promoted the activation of PPARγ in macrophages (Zhang et al., 2012) and facilitated the nuclear translocation of PPARα in fat-fed apoE^−/−^ mice, thus exerting beneficial effect on atherogenesis (Li et al., 2016). Therefore, it offers an original possibility that H_2_S might induce the expression of ACE2 in endothelial cells via modulating the activation of PPARα. Moreover, latest studies reported that H_2_S participated in regulating the expression of microRNAs (miR-129, miR-299b, and miR-369) in Ang II-induced hypertensive kidney (Weber et al., 2017). Na_2_S, an H_2_S donor alleviated ischemic and inflammatory injury in cardiomyocytes through upregulation of miR-21 (Toldo et al., 2014). Epigenetic modulation of miRNAs raises another possible way that H_2_S may post-transcriptionally regulate ACE2 expression through miRNAs in atherosclerosis. To further prove these assumptions and elucidate the precise mechanism for the induction of ACE2 expression by H_2_S, more research is warranted.

In addition to endothelial cells, macrophages are the main source of proinflammatory mediators and contribute to the initiation and development of atherosclerosis. Previous studies have implied the regulatory role of H_2_S in monocyte/macrophage activation (Zhi et al., 2007; Zhang et al., 2012). Here, we further investigated whether H_2_S affected the function of macrophages involving the pathway of ACE2-Ang-(1-7). It was found that NaHS significantly suppressed the production of TNF-α and MCP-1 in RAW264.7 cells. The anti-inflammatory property of NaHS was not reversed by pretreatment with DX 600. These findings indicate that H_2_S possibly inhibited the activation of macrophages through other pathways, but not ACE2 dependent pathway. Clearly, further studies are needed to elucidate the correlation between H_2_S and RAS in macrophages. In contrast to the anti-inflammatory effect of H_2_S observed in the present study, some researchers demonstrate that H_2_S acts as a pro-inflammatory mediator in sepsis and acute pancreatitis (Bhatia et al., 2005; Zhang et al., 2006). H_2_S stimulated the synthesis of pro-inflammatory cytokines in human monocyte cell line (Zhi et al., 2007). The discrepancy suggests that H_2_S plays various roles in different inflammatory conditions. Different cell types and animal models used in these studies may contribute to this divergence.

ACE is known to assist the conversion from Ang I to Ang II while ACE2 is a major enzyme to degrade Ang II to Ang-(1-7). Here, H_2_S was found to have negligible effect on the mRNA expression of ACE in endothelial cells. Thus, H_2_S regulated the carotid level of Ang II mainly by local ACE2. However, some studies proposed the role of H_2_S in regulating ACE-Ang II-AT1R axis. H_2_S inhibited Ang II/AT1R pathway and improved endothelial function and myocardial remodeling in renovascular hypertensive rats (Xue et al., 2015; Liu et al., 2017). NaHS also inhibited hyperglycemia-induced ACE-Ang II-AT1R activation in cultured renal mesangial cells and kidneys from diabetic rats (Xue et al., 2013). The discrepancy about the role of H_2_S in regulating ACE may be due to different animal models and cell lines utilized in experiments. Future work is needed to clarify this divergence and expand the understanding of the effect of H_2_S on RAS.

In conclusion, our findings propose deficiency of endogenous H_2_S formation as well as downregulation of ACE2 in carotid atherosclerosis induced by disturbed flow and high fat diet. Supplement of H_2_S promotes ACE2 expression and production of Ang-(1-7) in endothelial cells, resulting in attenuation of atherosclerosis.

## Author contributions

HZh and CW supervised the whole project. YL, HZe, and LG performed the major experiments. TG provided the technical support.

### Conflict of interest statement

The authors declare that the research was conducted in the absence of any commercial or financial relationships that could be construed as a potential conflict of interest.

## Supplementary material

Figure S1 Representative sections used for morphometric analysis. Sections were measured by image analysis stained with hematoxylin and eosin (H&E) and Masson's trichrome. Arrowheads indicated external elastic lamina (EEL). Small arrows indicated internal elastic lamina (IEL).

Figure S2 Effect of NaHS on Mas, a functional receptor for Ang-1-7 and ACE expression in unstimulated HUVECs or HUVECs stimulated with LPS (100 ng/ml). HUVECs were pre-incubated with saline or NaHS (100 μM) for 24 and then stimulated with saline or LPS (100 ng/ml) for 24 h. Mas **(A)** and ACE **(B)** mRNA expression were analyzed by RT-PCR.

Figure S3 Effect of pretreatment with DX600, a selective ACE2 antagonist on suppressing the production of TNF-α nd MCP-1 induced by NaHS in unstimulated or LPS-stimulated murine macrophage cell line (RAW264.7). Before pre-incubation with NaHS (100 μM) for 24 h, RAW264.7 cells were pre-treated with DX600 (1 μM) for 2 h. Then the cells were stimulated with saline or LPS (100 ng/ml) for 6 h. Production of TNF-α **(A)** and MCP-1 **(B)** was assayed by ELISA. The data are means ± SEM of at least three independent experiments. ^*^*P* < 0.05 for the comparison between control at baseline and unstimulated HUVECs treated with NaHS. ^#^*P* < 0.05 for the comparison between control at baseline and LPS-stimulated HUVECs treated with saline. ^**^*P* < 0.05 for the comparison between LPS-stimulated HUVECs treated with saline and LPS-stimulated HUVECs treated with NaHS.
