# Supplementary figures and images for: Hydrogen Sulfide Attenuates Atherosclerosis in a Partially Ligated Carotid Artery Mouse model via Regulating Angiotensin Converting Enzyme 2 Expression
Source: Front Physiol. 2017 Oct 10;8:782. doi: 10.3389/fphys.2017.00782 (PMC5641337; doi:10.3389/fphys.2017.00782)

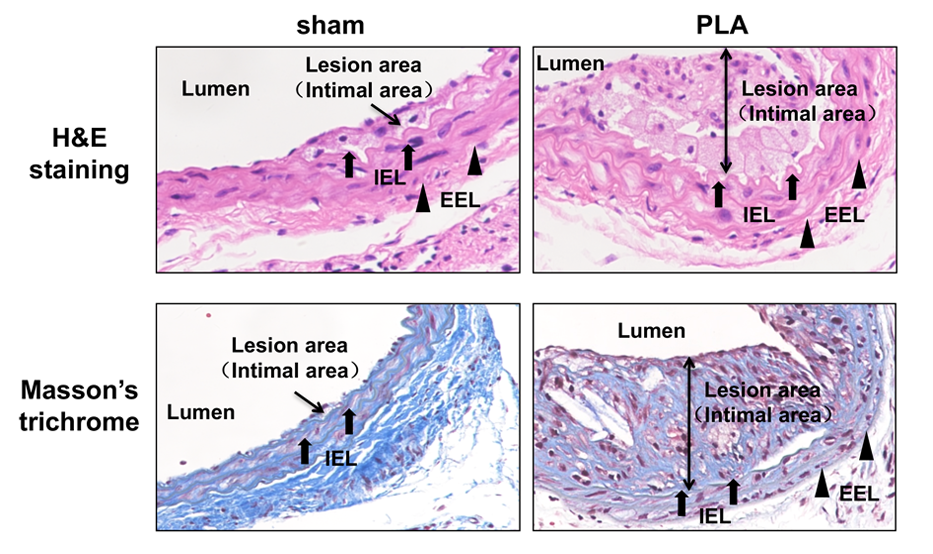

Supplement: Figure S1 — Representative sections used for morphometric analysis. Sections were measured by image analysis stained with hematoxylin and eosin (H&E) and Masson's trichrome. Arrowheads indicated external elastic lamina (EEL). Small arrows indicated internal elastic lamina (IEL). [file Image1.TIF]

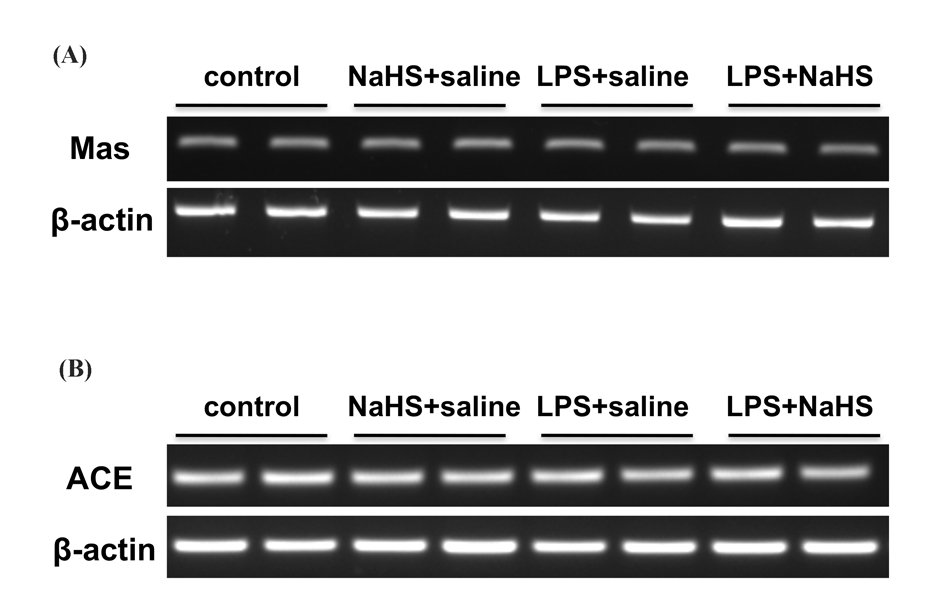

Supplement: Figure S2 — Effect of NaHS on Mas, a functional receptor for Ang-1-7 and ACE expression in unstimulated HUVECs or HUVECs stimulated with LPS (100 ng/ml). HUVECs were pre-incubated with saline or NaHS (100 μM) for 24 and then stimulated with saline or LPS (100 ng/ml) for 24 h. Mas (A) and ACE (B) mRNA expression were analyzed by RT-PCR. [file Image2.TIF]

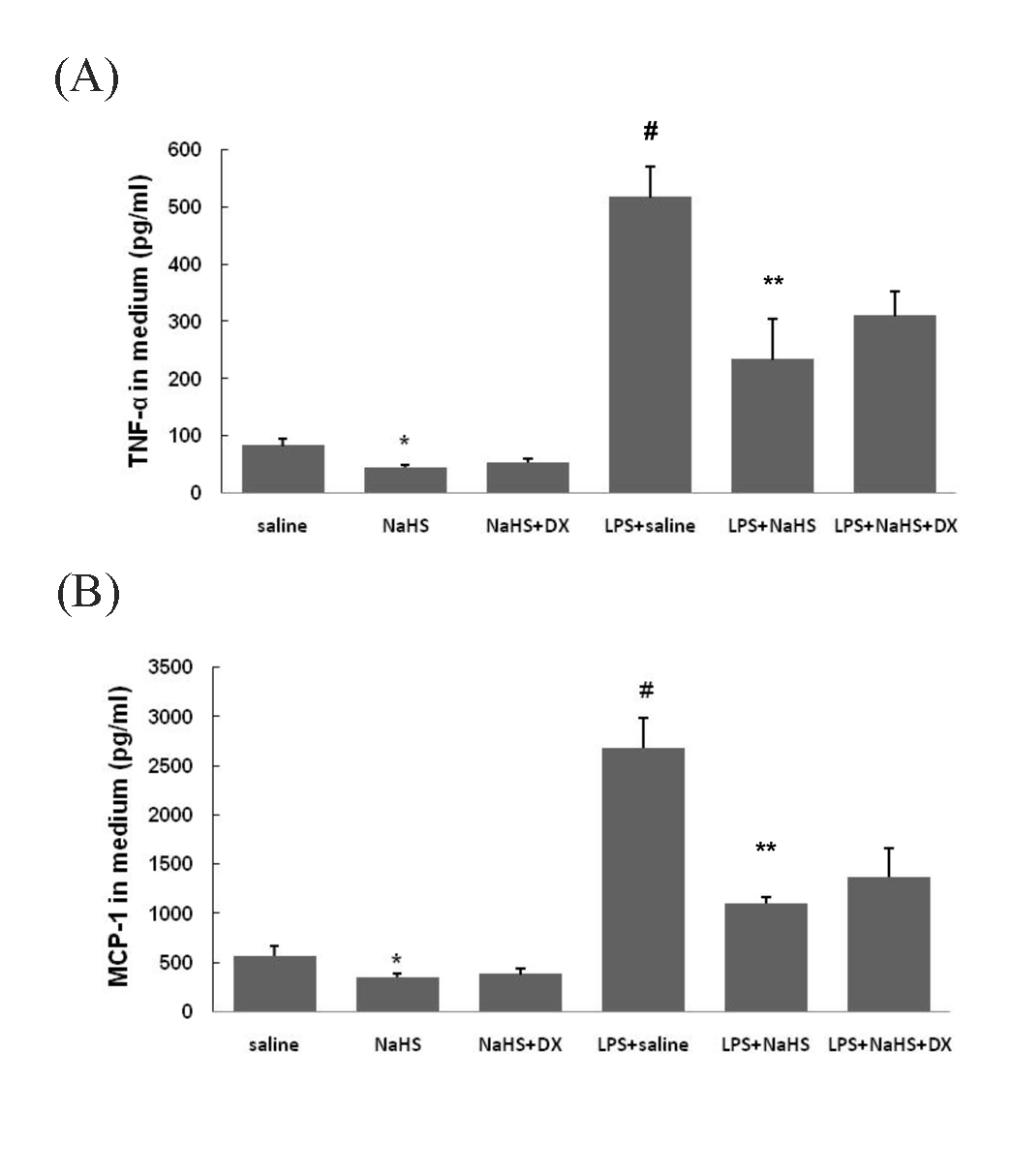

Supplement: Figure S3 — Effect of pretreatment with DX600, a selective ACE2 antagonist on suppressing the production of TNF-α nd MCP-1 induced by NaHS in unstimulated or LPS-stimulated murine macrophage cell line (RAW264.7). Before pre-incubation with NaHS (100 μM) for 24 h, RAW264.7 cells were pre-treated with DX600 (1 μM) for 2 h. Then the cells were stimulated with saline or LPS (100 ng/ml) for 6 h. Production of TNF-α (A) and MCP-1 (B) was assayed by ELISA. The data are means ± SEM of at least three independent experiments. *P < 0.05 for the comparison between control at baseline and unstimulated HUVECs treated with NaHS. #P < 0.05 for the comparison between control at baseline and LPS-stimulated HUVECs treated with saline. **P < 0.05 for the comparison between LPS-stimulated HUVECs treated with saline and LPS-stimulated HUVECs treated with NaHS. [file Image3.TIF]
